# Human Parechovirus 1 Infection Occurs via αVβ1 Integrin

**DOI:** 10.1371/journal.pone.0154769

**Published:** 2016-04-29

**Authors:** Pirjo Merilahti, Sisko Tauriainen, Petri Susi

**Affiliations:** Department of Virology, University of Turku, Turku, Finland; Faculty of Biochemistry Biophysics and Biotechnology and Malopolska Centre of Biotechnology, Jagiellonian University, POLAND

## Abstract

Human parechovirus 1 (HPeV-1) (family *Picornaviridae*) is a global cause of pediatric respiratory and CNS infections for which there is no treatment. Although biochemical and *in vitro* studies have suggested that HPeV-1 binds to αVβ1, αVβ3 and αVβ6 integrin receptor(s), the actual cellular receptors required for infectious entry of HPeV-1 remain unknown. In this paper we analyzed the expression profiles of αVβ1, αVβ3, αVβ6 and α5β1 in susceptible cell lines (A549, HeLa and SW480) to identify which integrin receptors support HPeV-1 internalization and/or replication cycle. We demonstrate by antibody blocking assay, immunofluorescence microscopy and RT-qPCR that HPeV-1 internalizes and replicates in cell lines that express αVβ1 integrin but not αVβ3 or αVβ6 integrins. To further study the role of β1 integrin, we used a mouse cell line, GE11-KO, which is deficient in β1 expression, and its derivate GE11-β1 in which human integrin β1 subunit is overexpressed. HPeV-1 (Harris strain) and three clinical HPeV-1 isolates did not internalize into GE11-KO whereas GE11-β1 supported the internalization process. An integrin β1-activating antibody, TS2/16, enhanced HPeV-1 infectivity, but infection occurred in the absence of visible receptor clustering. HPeV-1 also co-localized with β1 integrin on the cell surface, and HPeV-1 and β1 integrin co-endocytosed into the cells. In conclusion, our results demonstrate that in some cell lines the cellular entry of HPeV-1 is primarily mediated by the active form of αVβ1 integrin without visible receptor clustering.

## Introduction

Integrins are heterodimeric transmembrane receptor proteins that mediate cell-cell and cell-extracellular matrix (ECM) interactions [1] often via a specific arginine—glycine—aspartic acid (RGD) motif. RGD-binding integrins include five αV integrins (αVβ1, αVβ3, αVβ5, αVβ6, and αVβ8), two β1 integrins (α5β1 and α8β1), and αIIbβ3 [2]. Human parechovirus 1 (HPeV-1) is one of the sixteen parechovirus types in the genus *Parechovirus* of the family *Picornaviridae* [3–15]. Parechovirus infections are commonly encountered during the first years of life and are often mild or asymptomatic [16–20]. However, besides gastroenteritis and respiratory infections, HPeV-1 causes infections of the central nervous system and severe generalized infections, as well as myocarditis especially in neonates [9,16,17,21,22]. The structure of a parechovirus is icosahedral, and like other picornaviruses, its genome is a positive-sense, single-stranded RNA molecule [23–25]. RGD motif resides on the surface of the HPeV-1 particle through which it interacts with cell surface integrin receptor(s) [26]. Among human picornaviruses, there are ten virus types that possess the RGD motif within the VP1 protein, but integrin binding has been shown experimentally only for coxsackievirus A9 (CV-A9), echovirus 9 (E-9), echovirus 1 (E-1), and HPeV-1 [26]. Remarkably, all cultivable parechoviruses with the exception of HPeV-3, possess the RGD motif suggesting that they all may bind and use integrin receptor(s) during infectious entry.

HPeV-1 has been shown to bind *in vitro* to αVβ1, αVβ3 and αVβ6 integrins [27–29], while it has been reported that during cellular infection HPeV-1 favors αVβ3 over αVβ1 integrin [29]. HPeV-1 receptor binding and use have often been compared to a related picornavirus, coxsackievirus A9 (CV-A9), which also bears the RGD motif [26,30]. Whereas CV-A9 can infect some cell lines devoid of the RGD motif or cells that do not express αV integrins [31,32], HPeV-1 is more dependent on RGD-mediated integrin binding during cellular entry. After deletion of the RGD, the virus particles were essentially noninfectious, and only viruses in which the RGD sequence had been genetically restored were recovered [33]. We have recently shown that heparan sulfate possesses a role in HPeV-1 infection [34]. Another candidate receptor for HPeV-1 is matrix metalloproteinase 9 (MMP-9) [27], but these findings have not been corroborated by the others including us.

In the present study, we demonstrate that integrin αVβ1 plays a specific role in the infectious entry of HPeV-1 into A549, HeLa and SW480 cell lines. HPeV-1 did not bind to or internalize into β1 knock-out cell line (GE11-KO), whereas internalization into a cell line overexpressing β1 integrin (GE11-β1) was successful. HPeV-1 co-localized with β1 integrin on the cell surface and co-internalized into the GE11 cells. Activation of β1 integrin affected HPeV-1 infectivity but integrin receptor clustering was not detected.

## Materials and Methods

### Cells, viruses, and antibodies

Human cervical cancer (HeLa-Ohio), human colorectal adenocarcinoma (SW480), and human lung carcinoma (A549) cell lines were from the American Type Culture Collection (ATCC). The β1 knockout cell line GE11-KO and its derivative β1 overexpressing cell line, GE11-β1 (GE11- β1A), were kind gifts from Arnoud Sonnenberg (The Netherlands Cancer Institute, The Netherlands) [35]. The cells were maintained in Dulbecco’s modified Eagle Medium (DMEM) supplemented with 10% fetal calf serum (FCS) and gentamicin. In experiments where antibodies were used, DMEM was supplemented with 1 mM MgCl_2_.

HPeV-1 (prototype, Harris strain) [3] and CV-A9 (prototype, Griggs strain) [30,36] were propagated in A549 cells and purified in sucrose gradients as described earlier [32]. Clinical HPeV-1 isolates with low passage numbers were from Dr. Katja Wolthers (Academic Medical Center, The Netherlands). The culture medium for virus infections was supplemented with 1% FCS, and the efficiencies of virus infections were determined with plaque titration assays.

The function blocking β1 integrin antibody 6S6 (MAb 2253) was from Millipore and the β1 integrin activating antibody TS2/16 (sc-53711) was from Santa Cruz. Other cell surface antibodies used were α5β1 (MAb 1969, Millipore) and αV (L230, ATCC) which both are integrin function blocking antibodies. Fluorescence conjugated antibodies against β1 (303015) and αV (327907) integrins, both from BioLegend, as well as unconjugated primary antibodies against αVβ3 (MAb 1976), αVβ6 (MAb 2077z) and α5β1 (MAb 1999), all from Millipore, were used in the flow cytometry analysis. Alexa Fluor (AF) 488-, 568- and 633-labeled anti-mouse and anti-rabbit secondary antibodies and 568-labeled phalloidin were from Life Technologies. Hoechst 33342 for staining nuclei was from Sigma-Aldrich. Samples for confocal microscopy were mounted with Prolong Gold anti-fade Reagent containing DAPI (Life Technologies). HPeV-1 and CV-A9 antisera were obtained by immunizing rabbits with sucrose gradient-purified viruses as described previously [37–39].

### Flow cytometry

Monoclonal antibodies were used to detect αV, β1, αVβ3, αVβ6 and α5β1 integrins on A549, HeLa and SW480 cell lines, and the expression level of β1 integrin was analyzed on GE11-KO and GE11-β1 cells. The detached cells were suspended in the buffer solution (PBS containing 0.5% BSA), which was used throughout the experiment. Primary antibodies were added into the buffer and incubated for 1 h at 4°C. The cell pellets were washed and incubated with the secondary antibodies for 1 h at 4°C, and after washing the cells were suspended in the buffer. Flow cytometry measurements were done with a FACSCalibur flow cytometer (Becton Dickinson) and in total, 20 000 cells were analyzed in each experiment. The results were processed with a flow cytometry data analysis software (Flowing Software, www.flowingsoftware.com).

### HPeV-1 internalization and infectivity assays

Cells (A549, HeLa and SW480) were cultivated on 96-well plates (PerkinElmer) and inoculated with HPeV-1 at MOI of 10. After incubation for 1 h on ice, unbound viruses were removed, a pre-warmed infection medium was added, and the cells were transferred to a 37°C CO_2_ incubator. After 6 h incubation, the cells were washed with PBS, fixed (15 minutes with 4% formaldehyde in PBS), permeabilized (10 minutes with 0.1% Triton-X100 in PBS) and stained (antibody dilutions were made in 3% BSA in PBS) with a virus-specific antiserum, and AF 488-labeled secondary antibodies at RT for 1.5 h. The nuclei were stained with Hoechst and infection efficiency was visualized by fluorescence microscope using a Zeiss Axiovert 200M (10 or 20× objective) (Zeiss).

### Antibody blocking assay

15 μg /ml of function blocking antibodies in serum free DMEM, containing 1 mM MgCl_2_, were added onto confluent SW480 cells in 96-well plates and incubated for 1 h in RT with gentle shaking. Unbound antibodies were washed away with PBS and the cells were infected at MOI of 1 similarly as in the infectivity assay, stained with HPeV-1-specific antiserum and secondary antibody, and detected with a Zeiss Axiovert 200M (10× objective) microscope. The success rate (percentage of infected cells) was calculated with BioImageXD imaging software [40] (www.bioimagexd.net) as described below. The cells infected in the absence of blocking antibodies were used as a mock control (100% infection), and the mean of duplicates of three experiments (more than 10 000 cells per antibody) was calculated. P-values were analyzed with paired t-test.

### Internalization of HPeV-1 and CV-A9 into GE11-KO and GE11-β1 cells

GE11-KO and GE11-β1 cells were grown on cover slips in 24-well plates and HPeV-1 (Harris), clinical HPeV-1 isolates, and CV-A9 (Griggs) as a control were inoculated onto cells. Viruses were incubated with the cells for 1 h on ice, after which unbound viruses were washed with PBS. Pre-warmed infection medium was added and incubated at 37°C for 6 h. The cells were washed, fixed and permeabilized as described above. For immunofluorescence labeling, the antibodies were diluted in PBS containing 3% BSA, and the cells were incubated with the antibodies at room temperature, primary antibodies for 1 hour and secondary antibodies for 30 minutes. The actin filament stain, phalloidin, was added with secondary antibodies to determine the borders of the cells. Following washing with PBS, the cells were mounted with Prolong Gold Antifade Reagent and examined with a Zeiss LSM780 confocal microscope using Plan-Apochromat objectives (63× / 1.2 oil/water for HPeV-1 imaging, and 40×/ 1.2. oil/water for CV-A9 imaging).

### Activation of integrins

SW480 cells were treated with β1 integrin activating antibody (TS2/16) at different concentrations (5 μg, 20 μg, and 50 μg /ml) in 96-well plates and incubated at 37°C for 1 h. After incubation, HPeV-1 infection and virus staining was performed as described above in the infectivity assay. The samples were analyzed with a Zeiss Axiovert 200M (10× objective) and BioImageXD software. The mock treated cells were used as a control (100% infection) and the mean was calculated from four parallel samples (20 000–40 000 cells/antibody concentration). P-values were analyzed with paired t-test.

### Integrin clustering

SW480 cells were grown on cover slips in 24-well plates. The experimental procedure was performed as described in echovirus 1 studies [41,42]. Samples included 1) a negative control for clustering, where cells were incubated only with primary β1 integrin antibody (TS2/16) followed by fixing and staining with a secondary antibody and DAPI, 2) a positive control for clustering, where cells were first incubated with β1 integrin antibody, after which the secondary antibody was added to induce β1 integrin clustering, followed by fixing and DAPI staining, and 3) a virus sample, where cells were incubated with HPeV-1 for 15 minutes followed by fixing and staining (anti-HPeV-1, anti-β1 integrin antibodies and DAPI for staining nuclei). The samples were examined with a Zeiss LSM780 confocal microscope using a Plan-Apochromat objective (63× / 1.2 oil/water).

### HPeV-1 co-localization with β1 integrin in GE11-β1 cells

To test HPeV-1 co-localization with β1 integrin, GE11-β1 cells were inoculated with HPeV-1 at MOI of 5. After 1 h on ice, unbound viruses were removed, a pre-warmed infection medium was added, and the cells were transferred to 37°C where the infection was followed. After specific time points (0, 5 and 30 minutes post infection) the cells were washed, fixed and permeabilized. However, the 0-min sample was not permeabilized because the co-localization was analyzed from the cell surface only. The samples were stained as described earlier and examined with a Zeiss LSM780 confocal microscope using a Plan-Apochromat objective (63× / 1.2 oil/water). Co-localization analyses (automatic thresholding after background subtraction, Costes P-value calculation with 100 iterations) of selected image stacks were performed with BioImageXD software [40].

### BioImageXD analysis

The calculation of infection efficiency was analyzed with BioImageXD software [40]. Image datasets taken with Zeiss Axiovert 200M were imported and the total number of cells (blue channel, nuclei) and infected cells (green channel, HPeV-1) were calculated separately, after which the percentage of infection was calculated manually. The settings were set on the basis of control cells, and image threshold was set to make all cells visible based on staining of nuclei (blue channel). The number of objects was then automatically calculated. The analysis was performed in the same way for the infected cells but in this case a green channel was used indicating only the cells positive for virus staining. The percentage of infections was calculated manually after determining the numbers of infected and total cell count.

## Results

### HPeV-1 infects cells in the absence of integrin αVβ3 and αVβ6

Previous studies indicate that both human parechovirus 1 (HPeV-1) and a related enterovirus, coxsackievirus A9 (CV-A9), bind to integrin αVβ3 and αVβ6 *in vitro* [28,31,43–45]. It has also been suggested that HPeV-1 may use either αVβ1 or preferentially αVβ3 integrin as cellular receptor [29]. To elucidate the role of integrin αVβ1, αVβ3, and αVβ6 in HPeV-1 infection *in vivo*, the receptor tropism of HPeV-1 in three cell lines, A549, HeLa, SW480, was examined.

**Fig 1:**
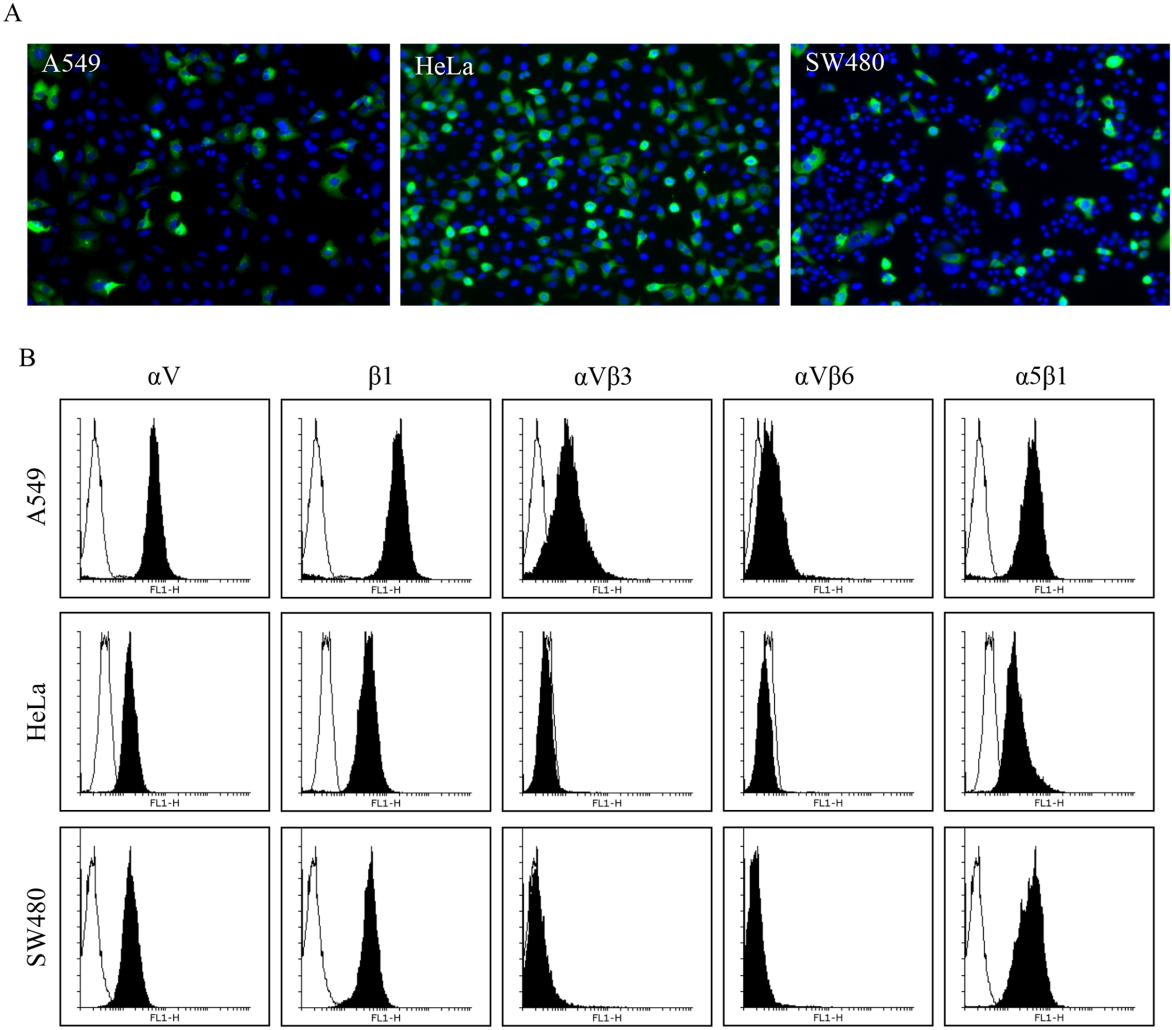
HPeV-1 infects the cell lines that do not express αVβ3 and αVβ6 integrins. (A) A549, HeLa, and SW480 cell lines were inoculated with HPeV-1 at MOI of 10, unbound viruses were removed followed by incubation at 37°C for 6 h, and processed for immunofluorescence staining and imaging (with a 20×objective). HPeV-1 particles and nuclei are shown in green and blue, respectively. (B) The receptor profile of A549, HeLa and SW480 cells was analyzed with flow cytometry. Cells were fixed, and cell surface integrins (αV, β1, αVβ3, αVβ6 and α5β1) were stained using specific antibodies, and 20 000 cells/antibody was measured by flow cytometry.

Firstly, we determined the susceptibility of these three cell lines for HPeV-1 infection. The cells were infected at MOI of 10, washed after one hour binding on ice, and the infection was followed for six hours before staining with HPeV-1 specific antiserum. The infection was visualized by immunofluorescence microscopy, and the results show that HPeV-1 internalizes and infects these cell lines efficiently (Fig 1A). Secondly, we performed a flow cytometry analysis which showed, as expected, that A549 cells express integrin subunits αV and β1 as well as integrin αVβ3 and αVβ6 while expression of αVβ3 and αVβ6 is not detected on HeLa and SW480 cells (Fig 1B). All three cell lines highly express integrin α5β1. Both αV and β1 are shown on the cell surface with high levels suggesting that integrin αVβ1 is available for virus binding. The visualization of the αVβ1 heterodimer was performed with subunit specific antibodies because specific antibodies against integrin αVβ1 are not commercially available. The results indicated that irrespective of the integrin expression profile, all these cell lines supported HPeV-1 replication based on the immunofluorescence images and RT-qPCR at 1 h and 6 h time points (S1 Fig). No virus was visualized at 0 h and 1 h time points whereas virus accumulation was evident at 6 h time point. This suggested that neither αVβ3 nor αVβ6 is required for efficient HPeV-1 infection into A549, HeLa and SW480 cells.

### HPeV-1 utilizes αVβ1 integrin in human epithelial colon carcinoma cell line

**Fig 2:**
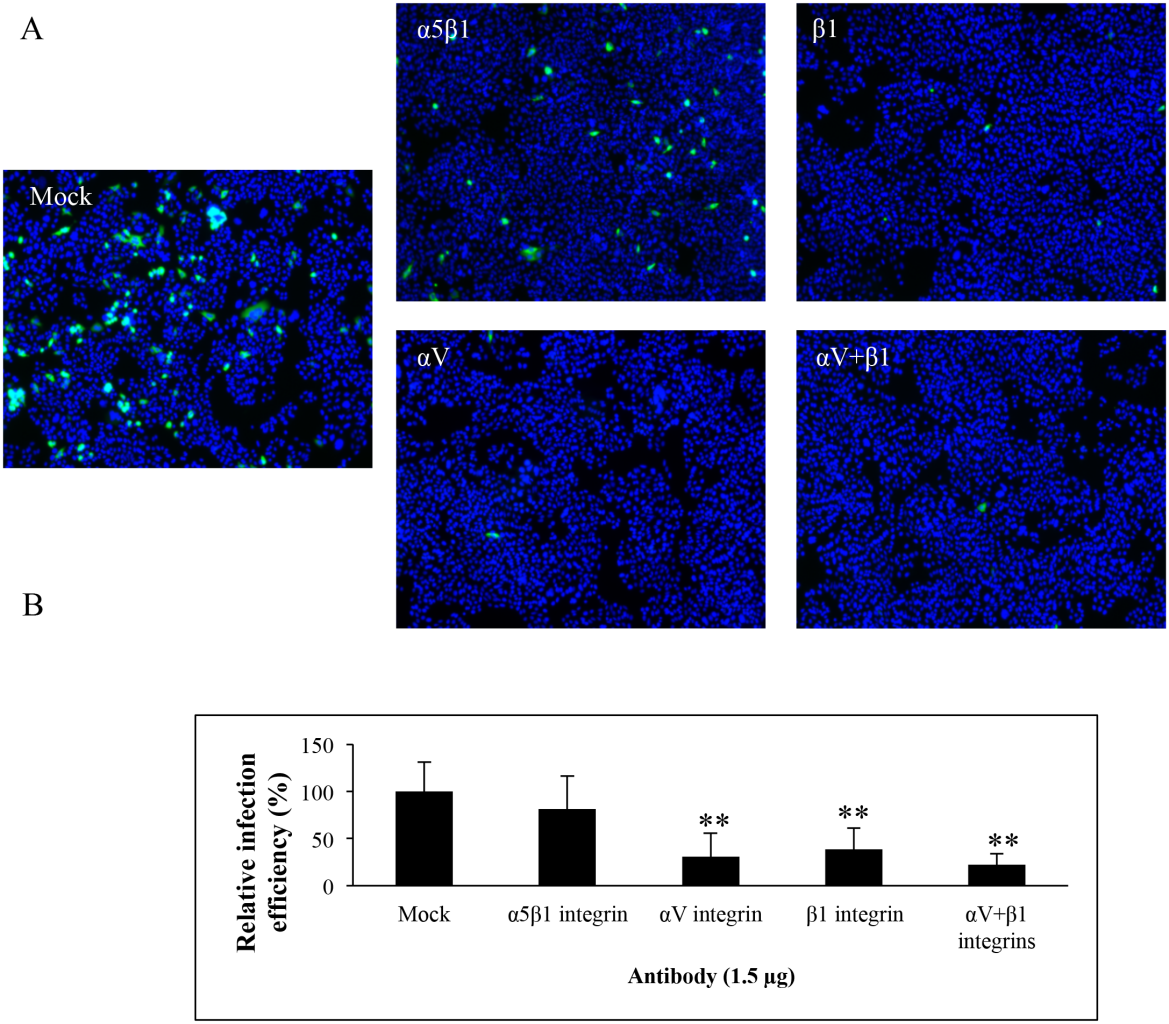
HPeV-1 infection is inhibited by integrin function-blocking antibodies. (A) Immunofluorescence microscopy of antibody-treated SW480 cells infected with HPeV-1. SW480 cells were treated with 15 μg/ml of anti-integrin antibodies for 1 h and subsequently infected with HPeV-1 at a MOI of 10. The cells were incubated with HPeV-1 for 1 h on ice followed by wash and incubation for 6 h at 37°C. The cells were fixed and stained with nuclear stain Hoechst (blue) and HPeV-1 specific antiserum (green). (B) Relative infection efficiency of HPeV-1 in integrin-treated cells. SW480 cells were processed as in Fig 2A. The infection efficiency of HPeV-1 in total of 10 000 cells was counted from microscopic images, and p values were calculated. Percentage of HPeV-1 infection in mock-treated (control) cells was set as 100%. Standard deviations are shown, and ** indicates p<0.001. The cells were imaged with a 10×objective.

We and others have used a human epithelial colon carcinoma SW480 cell line in CV-A9 infection studies, and shown that CV-A9 infection proceeds independent of αV integrin [45]. Because HPeV-1 and CV-A9 have similar *in vitro* integrin-binding properties [28,31], the dependency of HPeV-1 on αV integrin was studied further by using integrin function-blocking antibodies (Fig 2). SW480 cells were blocked with αV and β1 integrin antibodies and with α5β1 integrin antibody. Currently, commercial αVβ1 specific antibodies are not available, and therefore anti-αV and anti-β1 antibodies were used separately and in combination. Cells were incubated with function blocking antibodies prior to HPeV-1 infection, stained, and imaged with a fluorescence microscope. Relative percentages of HPeV-1 infections were calculated using BioImageXD software (Fig 2), and infection efficiency of mock treated SW480 cells was set to 100 percent. As shown in Fig 2B, there was 70% (p = 2.66 ×10^−4^) and 60% (p = 7.37×10^−4^) reduction in relative infection efficiency in anti-αV and anti-β1 antibody-blocked cells, respectively, compared to non-blocked control, while the combined effect of αV and β1 antibodies resulted in almost 80% (p = 3.07×10^−7^) reduction in HPeV-1 infectivity. Because α5β1 integrin is also capable of binding the RGD motif and functions similarly to αVβ1 [46], its ability to block HPeV-1 was also tested. The reduction caused by the blocking α5β1 integrin antibody was in the range of 20%, which suggests together with the other data that integrin αVβ1, and not α5β1, is the principal receptor for infectious entry of HPeV-1 into SW480 cell line.

### β1 integrin is required for HPeV-1 internalization into GE11 cells

**Fig 3:**
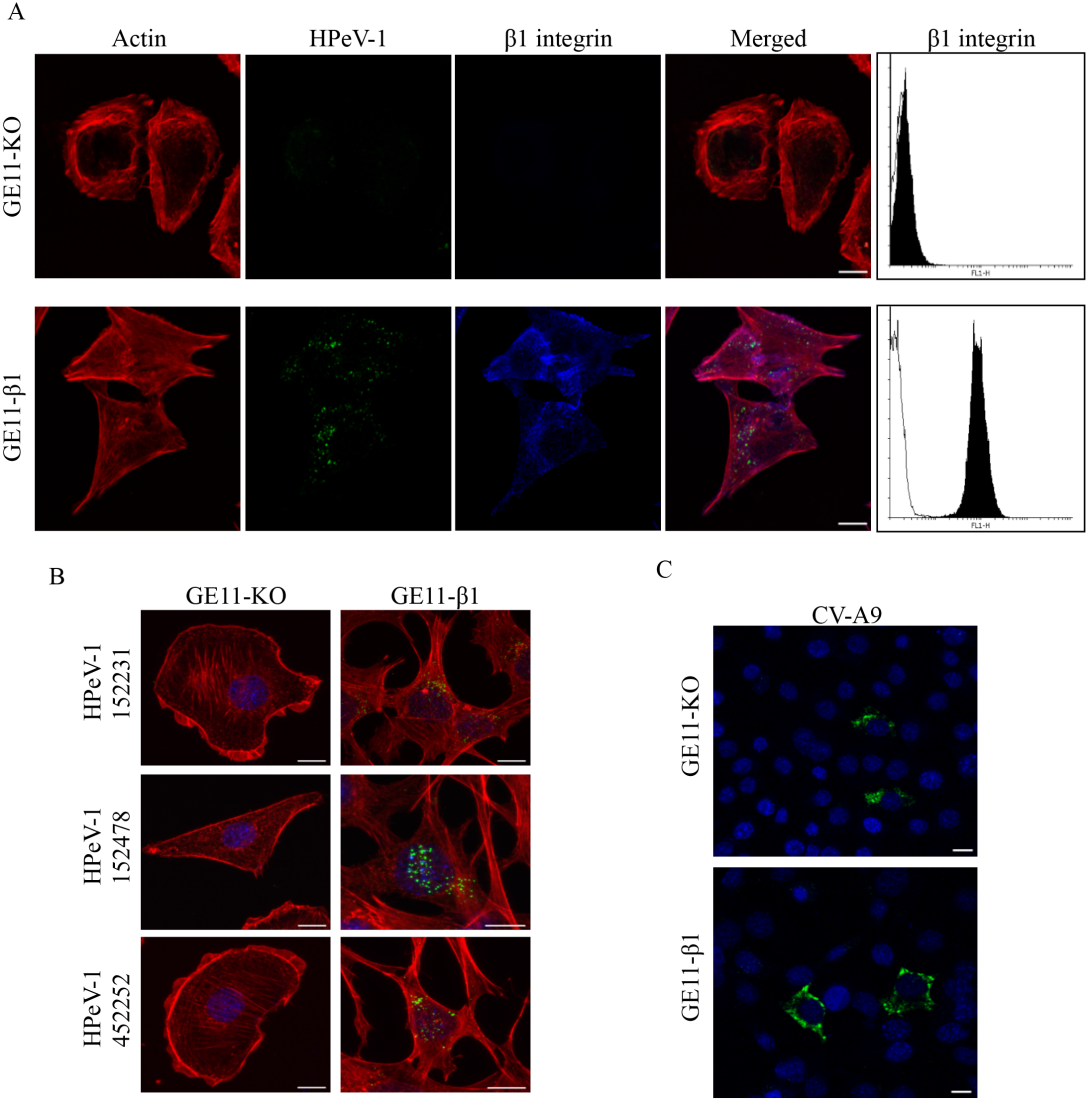
HPeV-1 internalization is inhibited in mouse cells lacking β1 integrin expression. (A) HPeV-1 internalizes into GE11-β1 but not GE11-KO cells. GE11-KO and -β1 cells were inoculated with HPeV-1 at a MOI of 5 on ice for 1 h followed by incubation for 6 h at 37°C. The cells were fixed, permeabilized, and stained with appropriate stains or antibodies as described in Materials and Methods. Actin filaments are shown in red, HPeV-1 in green and β1 integrin in blue. The β1 integrin expression on the cell surface was also analyzed by flow cytometry (right panel). (B) Clinical HPeV-1 isolates with low passage numbers (152231, 152478 and 452252) act similarly to the prototype. The cells were treated as in (A). (C) CV-A9 infects both GE11-KO and GE11-β1 cells. The CV-A9 infection was performed similarly to the HPeV-1 assay, followed by staining with anti-CV-A9 antibody (green) and DAPI (blue). Microscopic imaging was performed with Zeiss LSM780 confocal microscopy using a Plan-Apochromat objectives (63× / 1.2 oil/water [panels A and B] or with 40×/ 1.2. oil/water [panel C]). Bar 10 μm.

Previous studies with receptor use of HPeV-1 have been performed either using purified integrins in *in vitro* experiments [28], or by measuring virus binding to cell surface using cell sorting and immunoprecipitation techniques [29,44]. It can be speculated whether *in vitro* binding represents the actual situation during dynamic cellular infection, whereas cell sorting experiments do not exclude the possibility that other receptors than integrins are included in the entry process. We therefore used a knock-out cell line to examine the use of β1 integrin as the receptor of HPeV-1 [35]. The HPeV-1 infectivity assay was performed in a mouse epithelial cell line, GE11-KO, with genetic knock-out of β1 gene [35]. As a control, the same cell line overexpressing the β1 subunit, GE11-β1 [35], was used (Fig 3A). The expression of β1 integrin was confirmed by flow cytometry (Fig 3A, right panel) and also by staining, shown in blue in Fig 3A. Virus internalization into the GE11-β1 cell line was visualized by confocal microscopy, which allowed the detection of the virus within cell interior. S2 and S3 Figs show confocal slices of internalized HPeV-1 at 6 h and 24 h time points suggesting that virus accumulates but does not replicate or cause cytopathic effect in GE11-β1 cells. The data suggest that β1 integrin is essential for internalization of HPeV-1 into GE11-β1 cells.

We also tested the cellular tropism of three HPeV-1 clinical isolates, because it is possible that the HPeV-1 prototype (Harris strain), which dates back to 1950s, may not possess the characteristics of the recent HPeV-1 isolates. As shown in Fig 3B, the clinical isolates internalized into GE11-β1 but not into GE11-KO cells similarly to HPeV-1 Harris strain. We also showed that CV-A9 is capable of infecting both GE11-KO and GE11-β1 cells (Fig 3C), which is basically the first indication that these viruses have different cellular receptor tropism even though they have been shown to have similar *in vitro* binding properties to integrin αVβ3 and αVβ6 [28,31]. CV-A9 is clearly visible in the cytoplasm indicating that CV-A9 can replicate in these cell lines but this was not tested further. Although HPeV-1 cannot infect GE11-β1 cell line, the β1 integrin enables the HPeV-1 internalization. In the light of these findings, the data support the view that human β1 integrin possibly paired to mouse αV is sufficient to support HPeV-1 internalization in GE11 cells.

### Integrin activation mediates HPeV-1 infection without visible receptor clustering

**Fig 4:**
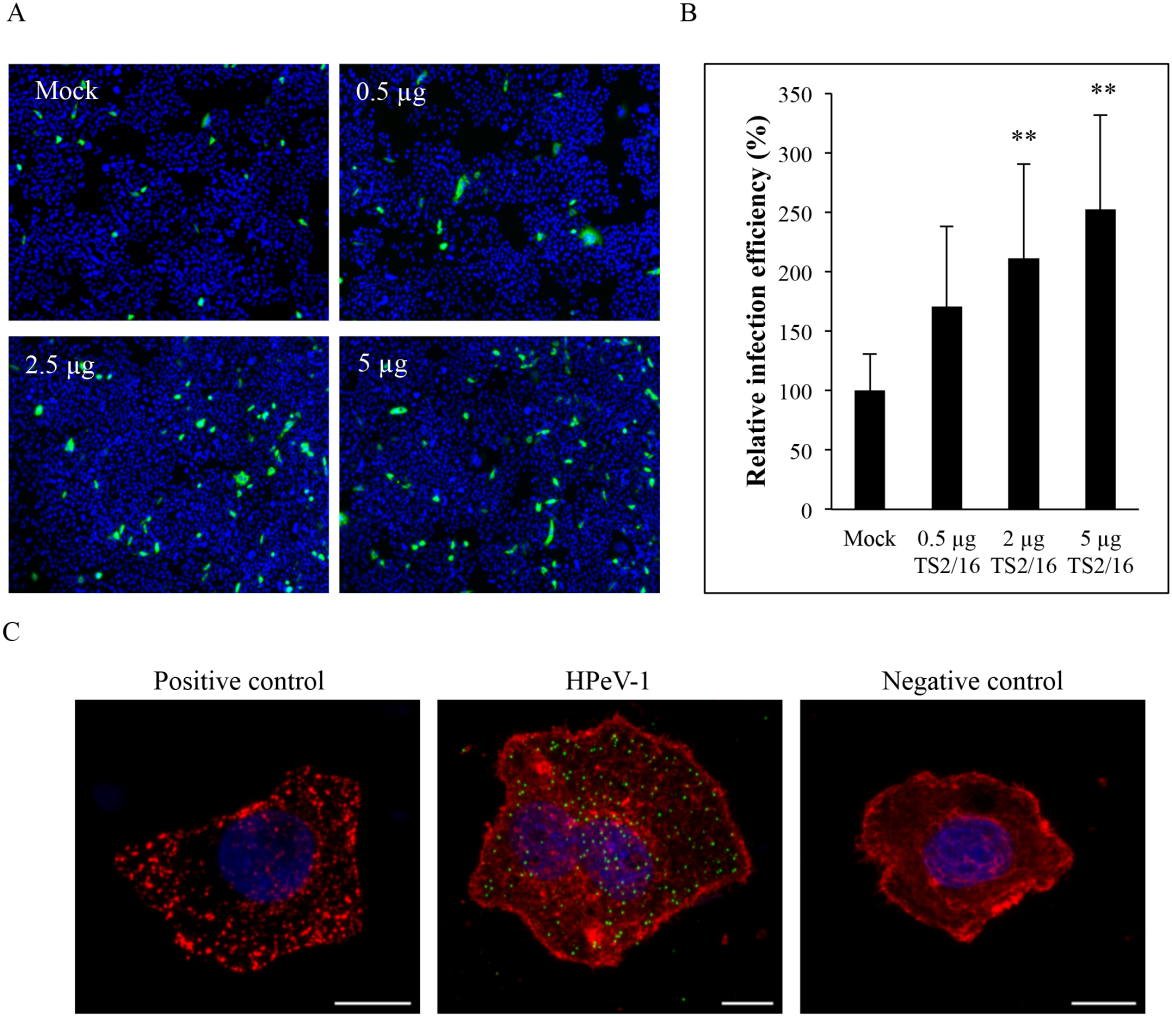
Activation of β1 integrins enhances HPeV-1 infectivity without visible receptor clustering. (A and B) SW480 cells were treated with β1 integrin activating antibody (TS2/16) at 37°C for 1 h after which the cells were inoculated on ice with HPeV-1 at a MOI of 10 followed by incubation for 6 h at 37°C. The cells were fixed, permeabilized and stained. The relative infection efficiency of HPeV-1 was calculated from nine parallel images (totally 20 000 to 40 000 cells per each antibody concentration) obtained with Zeiss Axiovert 200M (10×objective). The percentage of infection of HPeV-1 in mock-treated (control) cells was set as 100%. The error bars indicate standard deviation, * indicates p<0.01 and ** indicates p<0.001. (C) Receptor clustering is not detected during HPeV-1 infection in SW480 cell line. The cells were incubated with activating anti-β1 antibody for 15 min after which a secondary antibody was added, and incubation was continued for another 15 minutes before fixing of the cells (positive control, left panel). HPeV-1 was allowed to bind to SW480 cells for 15 minutes before fixing and staining (middle panel). SW480 cells were incubated with β1 primary antibody for 15 minutes prior to fixation of the cells. Integrin β1 is shown in red, HPeV-1 in green and nuclei in blue. Microscopic imaging was performed using Zeiss LSM780 confocal microscopy using a Plan-Apochromat objective (63× / 1.2 oil/water). Bar 10 μm.

Integrin activation is a controlled procedure by which the cell regulates ligand binding to integrins. It is sometimes followed by integrin receptor clustering, which is considered to be an important mechanism for generation of intracellular signals required for virus entry. The activation is known to be bi-directional: either from outside-in or inside-out. Extracellular domains of integrins are known to possess different conformations, which are highly dependent on the integrin type [47]. Previously, activating β1 integrin antibody TS2/16 has been shown to increase the entry rate of adenovirus 5 (Ad5) [48]. We used TS2/16 antibody to analyze the effect of integrin conformation on HPeV-1 infection and to test whether HPeV-1 infection is affected by integrin activation. SW480 cells were incubated for 1 h with different concentrations of TS2/16 antibody followed by HPeV-1 infection and staining. The cells were visualized by immunofluorescence microscopy (Fig 4A) and relative infectivity was measured from 20 000 to 40 000 cells with BioImageXD software. Infection of mock treated SW480 cells was set to 100 percent. Increasing the amount of activating β1-antibody increased the infectivity by 2.5-fold (Fig 4B). 0.5 μg of TS2/16 antibody increased infection to 170% (p = 1.96×10^−3^), 2 μg to 210 percent (p = 6.59×10^−5^) and 5 μg to 250% (p = 9×10^−7^) when compared to control. This indicates that the activation of β1 integrin affects HPeV-1 internalization and infection.

We also examined whether virus binding to cell surface induced integrin receptor clustering. Effective clustering of integrins has been demonstrated earlier with echovirus 1 (E-1, an enterovirus) [41,42], and we used the same protocol to study the clustering of β1 integrins during HPeV-1 infection (Fig 4C). A positive control, where β1 integrins have been clustered with β1 primary and secondary antibody prior to fixing, is shown on the left panel. HPeV-1 infected cell is shown in the middle, and a negative control where cells were incubated only with β1 antibody prior to fixing and staining is shown on the right. The β1 antibody-induced integrin clusters are clearly visible as bright spots around the cell. The infection of cells with HPeV-1 does not induce clear β1 integrin clustering, which is visible during E-1 infection [41,42]. The pattern of β1 integrins in infected cells looks similar to negative controls, where integrins are not clustered. However, it is possible that HPeV-1 causes small microclusters [49] that are not detectable at the resolution of light microscopy. It should be noted that because the cells were not permeabilized all visualized integrins and viruses are located on the cell surface and not inside the cell. The results showed that in SW480 cells, HPeV-1 does not lead to macroclustering of β1 integrins although virus infectivity is enhanced by the receptor activating antibodies.

### HPeV-1 co-localizes with β1 integrin during early stages of entry

**Fig 5:**
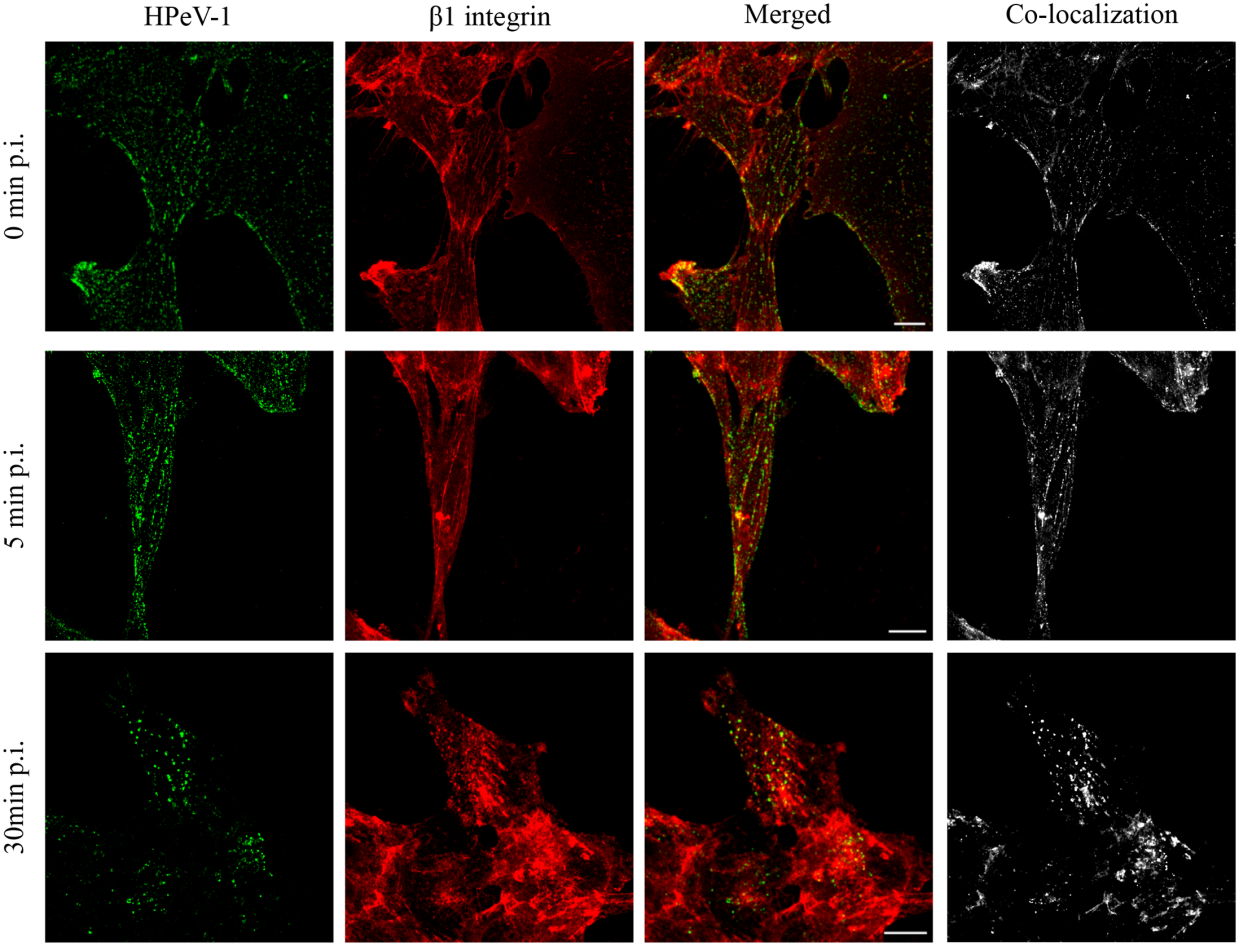
HPeV-1 co-localizes with β1 integrin during the early stages of virus entry. GE11-β1 cells were inoculated on ice for 1 h with HPeV-1 at a MOI of 5 and incubated for 0 (non-permeabilized), 5 and 30 minutes (permeabilized) at 37°C before fixing and staining. HPeV-1 is shown in green and β1 integrin in red. The right panel shows the co-localization analysis performed by BioImageXD software. The software calculates co-localizing voxels between two channels (HPeV-1 and β1 integrin), and these (co-localizing voxels) are shown in white. Microscopic imaging was performed with Zeiss LSM780 confocal microscopy using a Plan-Apochromat objective (63× / 1.2 oil/water). Bar 10 μm.

Successful HPeV-1 internalization in cell lines devoid of αVβ3 and αVβ6 integrins and in integrin β1 knock-out cell line overexpressing β1 suggested that HPeV-1 primarily uses the αVβ1 integrin during cellular infection. In GE11-β1 cells, HPeV-1 co-endocytose with β1 integrin during internalization and early stages of entry (Fig 5). We further analyzed co-localizations with BioImageXD software, which allows mathematical analysis of co-localization points, and thus gives more objective interpretation of the confocal images than visual inspection. The analyses with BioImageXD indicated that almost every virus particle was interacting with β1 integrin. This is illustrated in the right panel of Fig 5 in which all co-localizing voxels, which are located exactly in the same place in the sliced image, are shown in white. We also calculated Manders’ coefficient values (M1) of HPeV-1 co-localization to β1 integrin with BioImageXD. M1 values are proportional to the amount of fluorescence of the co-localizing objects in each component of the image, relative to the total fluorescence in that component. When M = 1.0, co-localization is fully achieved [50]. M1 of HPeV-1 infected samples were 1.0 (0 min), 0.84 (5 min), and 1.0 (30 min), P = 1.0. These data confirm that HPeV-1 binds to and is co-internalized with αVβ1 integrin.

## Discussion

Previously, human parechovirus 1 (HPeV-1) has been shown to interact with integrin αVβ1, αVβ3, or αVβ6 in *in vitro* binding assays or in a cryoEM reconstruction [28,29]. It has also been reported that during cellular infection HPeV-1 favors αVβ3 over αVβ1 integrin [29]. In this work, we analyzed the receptor-mediated internalization and infectious entry of HPeV-1 into A549 (human lung carcinoma), HeLa (human cervical cancer), SW480 (human colorectal adenocarcinoma), and mouse epithelial GE11 cell lines, and found that HPeV-1 internalized into cell lines that do not express integrin αVβ3 and αVβ6. Instead, αVβ1 integrin was shown to be essential for HPeV-1 entry.

To demonstrate the importance of β1 integrin in HPeV-1 internalization and infection, we used a β1-deficient mouse epithelial cell line GE11-KO [35]. Both prototype and clinical HPeV-1 isolates internalized into β1 overexpressing GE11 cells (GE11-β1), but not to GE11-KO. Thus, even if HPeV-1 did not replicate in GE11-β1 cells (S3 Fig), it is evident that β1 integrin is needed for efficient internalization of the virus. Previous study has shown that αV subunit, which is highly expressed in GE11-β1 cell line, pairs with β1 and β5 subunits [51]. However, it is unlikely that HPeV-1 uses αVβ5 as cellular receptor, because it does not support HPeV-1 infection in A549 cells [29]. An interesting result in our experiments was that whereas CV-A9 was capable of internalizing GE11-KO and GE11-β1 cell lines, HPeV-1 was not, which is basically the first demonstration that these viruses possess differential cellular receptor tropism. Previously, these viruses have been shown to have very similar *in vitro* binding properties to integrin αVβ3 and αVβ6 [28,31]. Both CV-A9 and HPeV-1 possess the RGD motif in the C-terminus of VP1 capsid protein [26,28,36], but only CV-A9 can infect cells even RGD motif was genetically removed [31]. However, the receptor for RGD-independent entry of CV-A9 remains unknown. In the light of the current findings it can be debated what is the actual role of RGD-binding integrins in the infectious entry of both CV-A9 and HPeV-1.

The observation that HPeV-1 internalizes into cells association with β1 integrin further supports the role of β1 as HPeV-1 receptor. At the zero time point, HPeV-1 co-localized completely with β1 integrin on the cell surface. Furthermore, HPeV-1 internalized into cells expressing β1 integrin, and these virus-integrin complexes accumulated in intracellular vesicles 30 minutes post-infection. To further demonstrate the specific role of β1 integrin in HPeV-1 infection, we used a β1 integrin activating monoclonal antibody, TS2/16, to activate β1 integrins. The activation of β1 integrins using this antibody prior to infection increased HPeV-1 infectivity, and this demonstrated that the virus may prefer the activated form of β1 integrin in cellular entry. This is different from echovirus 1 (E-1), which has been shown to bind to closed, non-activated, conformation of α2β1 [42]. Few other human viruses have also been reported to bind inactive integrin [52–55], but besides Adenovirus 5 (Ad5) [48], there seems to be no other human virus than HPeV-1 that specifically utilizes activated integrins in the cellular entry. However, binding of a related aphtovirus (within *Picornaviridae* family), Foot-and-mouth disease virus (FMDV), to β1 integrin has been shown to be affected by manganese ions and β1 integrin activating antibody 9EG7 [56]. Usually the activation of integrins induces intracellular signaling [1]. Virus-induced integrin signaling has been studied previously with few viruses, including cytomegalovirus [57–59], adenoviruses [60–62], herpesviruses [63,64], echovirus 1 [41,42,65], and coxsackievirus A9 [32]. It can be speculated that binding of HPeV-1 to active conformation of αVβ1 integrin mimics the binding of its natural ligands (fibronectin and vitronectin). However, αVβ1 integrin binding to its natural ligands occurs relatively weakly [46,66], which may provide HPeV-1 the opportunity to bind to its integrin receptor with higher affinity (or avidity) than their ligands, and this may increase the rate of infection.

One of the key events in virus-receptor interactions is the clustering of receptors, which may lead to signaling cascade facilitating virus internalization. It has not been demonstrated previously whether HPeV-1 binding to any integrin receptor induces clustering and cellular signaling. We used the anti-β1 TS2/16 antibody, because the same antibody could be used in both clustering of integrins and visualization of the cell-bound virus, i.e. the antibody did not interfere with virus binding to the β1 receptor. We did not see any integrin β1 clustering on the cell surface at the early stages (0 or 5 min p.i.) of HPeV-1 internalization. This suggested that HPeV-1 does not need to induce visible integrin clusters to be internalized. Integrin αVβ1 has been reported to localize predominantly diffused, and not in focal contacts, indicating that it does not interact with cytoskeletal proteins in the same manner as for example α5β1 integrin [46]. The fact that αVβ1 is randomly distributed in the cell instead of being clustered at specific locations may explain why HPeV-1 does not induce visible clusters (macroclusters) although we cannot exclude the possibility of microclustering. Our findings are opposite to E-1, an enterovirus, which binds to non-activated α2β1 integrin and induces clear receptor clustering [41,42]. Integrin clustering has also been studied with few other viruses. Studies with Ad5 have revealed that the location of RGD on penton protein affects virus binding to its integrin receptor (αVβ3) with subsequent clustering prior to internalization [67]. On the other hand, CV-A9 did not induce clustering of αVβ6 integrins during cell entry [32]. Recently, Vaccinia virus (VV) was shown to internalize into HeLa and GD25 cells via β1 integrin and by activating the PI3K/Akt signaling pathway [68], but it is not known if clustering was involved. Further studies are needed to elucidate the interplay between the lack of clustering and possible signaling events due to binding of HPeV-1 to (activated) integrin αVβ1.

Here, we suggest that HPeV-1 uses αVβ1 integrin, instead of αVβ3 and αVβ6 integrins, for cell internalization and cellular infection. Integrins are used as receptors by several viruses [69], and αVβ1 acts as a receptor for HPeV-1 and a few other viruses. Human metapneumovirus (hMPV) and Foot-and-mouth-disease virus (FMDV) utilize αVβ1 integrin [56,70], and Ad5 uses αVβ1 integrins as well as many other integrins as its receptors [48,71]. Integrin αVβ1 is also commonly expressed in many cancerous cell lines, and therefore it will be of interest to determine whether cancer cell lines other than those used in this study express αVβ1 and are susceptible to HPeV-1 infection [72,73]. In this regard, infection of HPeV-1 via β1 integrin suggests that it may be useful in oncolytic virotherapy. For example, human melanoma, breast cancer, and neuroblastoma cells highly express αVβ1 integrin [74–76], and these cells may be susceptible to cytolytic infection by HPeV-1. It remains to be determined whether small molecular compounds developed against integrin β1 and used in cancer therapy prove to be protective against parechoviral disease.

## Supporting Information

S1 Fig Infectivity assay controls. A549 control cells were treated, stained and imaged similarly to the other samples (Figs 1 and 2), but they were fixed at 0 min time point (A), 1 h time point (B) and 6 h time point (C). Control image (A) was negative for virus staining, which confirms that all green color in other images arises from internalized or newly-formed virus particles. At 1 h time point (B) was no visible staining, but at 6 h time point (C) green staining indicates replicated virus.
(PDF)

S2 Fig A single slice from stacked confocal image (from the Fig 3A). Virus particles shown in green are visible in the cell interior confirming that HPeV-1 internalizes the GE11-β1 cells.
(PDF)

S3 Fig HPeV-1 in GE11-β1 cells at 24 h post-inoculation. (PDF)

S1 Table Detection of HPeV-1 at 1 h and 6 h time points by RT-qPCR. Results are presented as the mean of Ct values from two parallel samples in the same run. In average, 3.3 difference in Ct values equals to 10-fold difference in the RNA amount in the original sample.
(PDF)
