# Supplementary figures and images for: Human Parechovirus 1 Infection Occurs via αVβ1 Integrin
Source: PLoS One. 2016 Apr 29;11(4):e0154769. doi: 10.1371/journal.pone.0154769 (PMC4851366; doi:10.1371/journal.pone.0154769)

A

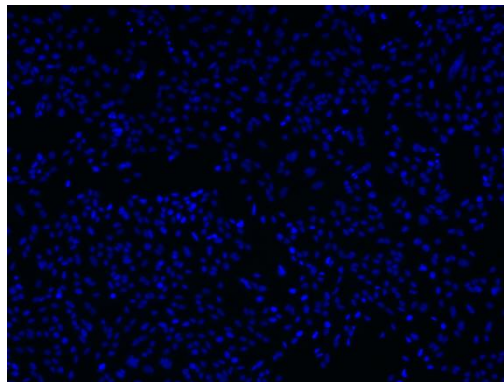

B

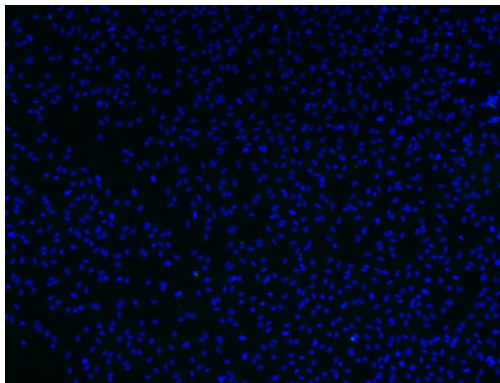

C

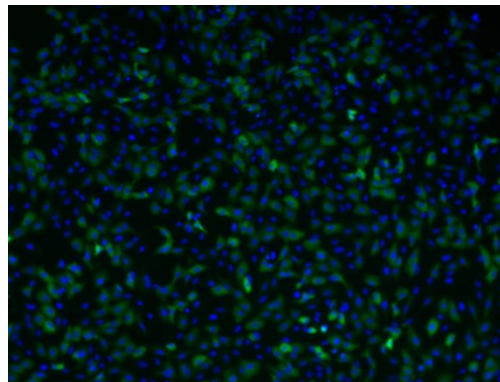

Supplement: S1 Fig — A549 control cells were treated, stained and imaged similarly to the other samples (Figs 1 and 2), but they were fixed at 0 min time point (A), 1 h time point (B) and 6 h time point (C). Control image (A) was negative for virus staining, which confirms that all green color in other images arises from internalized or newly-formed virus particles. At 1 h time point (B) was no visible staining, but at 6 h time point (C) green staining indicates replicated virus. (PDF) [file pone.0154769.s001.pdf]

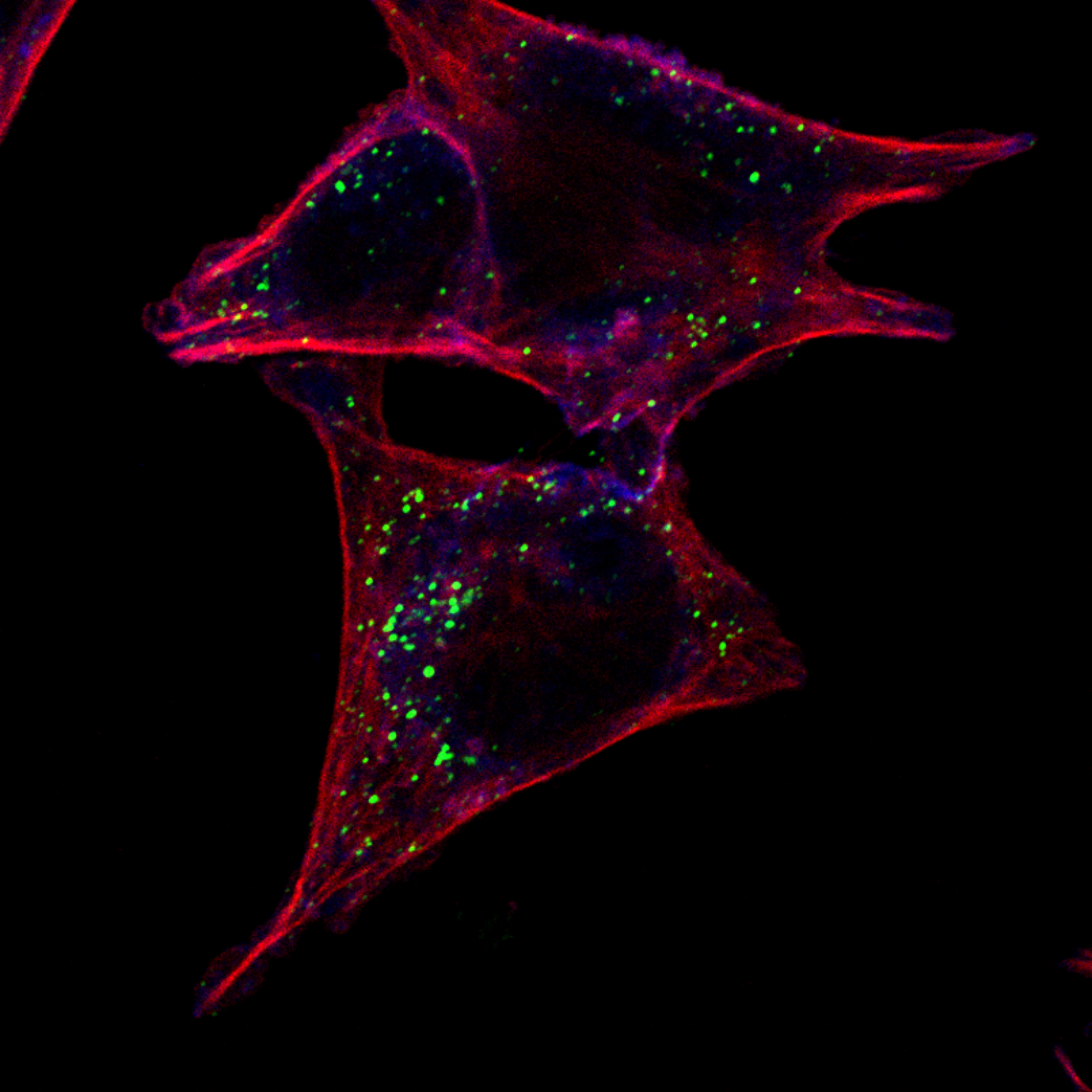

Supplement: S2 Fig — Virus particles shown in green are visible in the cell interior confirming that HPeV-1 internalizes the GE11-β1 cells. (PDF) [file pone.0154769.s002.pdf]

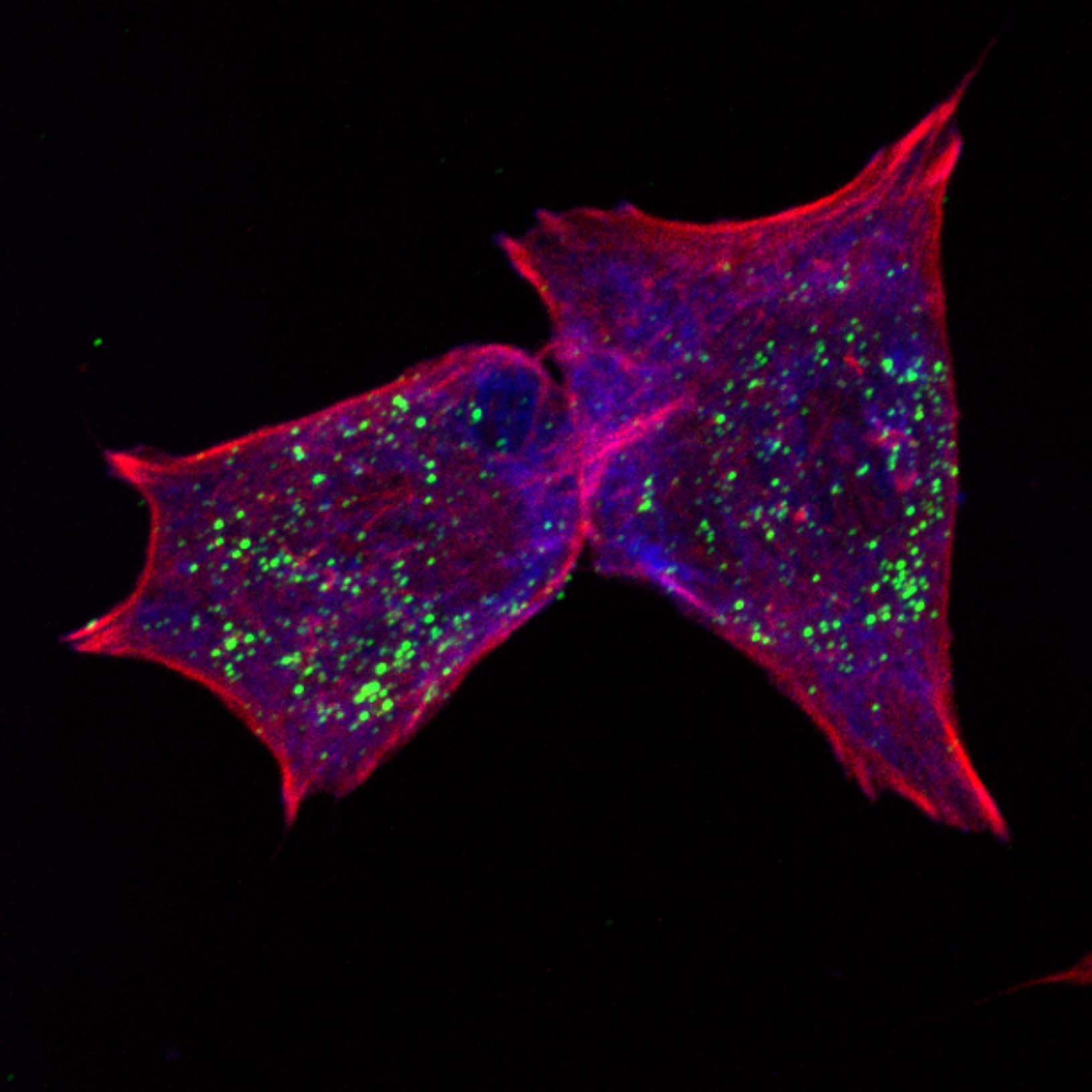

Supplement: S3 Fig — (PDF) [file pone.0154769.s003.pdf]
