# Supplementary material for: Human Parechovirus 1 Infection Occurs via αVβ1 Integrin
Source: PLoS One. 2016 Apr 29;11(4):e0154769. doi: 10.1371/journal.pone.0154769 (PMC4851366; doi:10.1371/journal.pone.0154769)
Supplement: S1 Table — Results are presented as the mean of Ct values from two parallel samples in the same run. In average, 3.3 difference in Ct values equals to 10-fold difference in the RNA amount in the original sample. (PDF) [file pone.0154769.s004.pdf]

S1 Table. Detection of HPeV-1 at 1 h and 6 h time points by RT-qPCR.

	Cell line		
Time point	A549	HeLa	SW480
1 h	34.4	41.5	41.8
6 h	30.1	38.5	34.1
